# Yttrium-Enriched Phosphate Glass-Ceramic Microspheres for Bone Cancer Radiotherapy Treatment

**DOI:** 10.1021/acsomega.4c02825

**Published:** 2024-12-16

**Authors:** Ben Milborne, Andi Arjuna, Md Towhidul Islam, Abul Arafat, Robert Layfield, Alexander Thompson, Ifty Ahmed

**Affiliations:** † Advanced Materials Research Group, Faculty of Engineering, University of Nottingham, Nottingham NG7 2RD, U.K.; ‡ School of Engineering, University of Wolverhampton, Telford Innovation Campus, Telford TF2 9NT, U.K.; § School of Life Sciences, Faculty of Medicine and Health Sciences, University of Nottingham, Nottingham NG7 2UH, U.K.; ∥ Biodiscovery Institute, Division of Cancer and Stem Cells, University of Nottingham, Nottingham NG7 2RD, U.K.; ⊥ Faculty of Pharmacy, Hasanuddin University, Makassar 90245, Indonesia

## Abstract

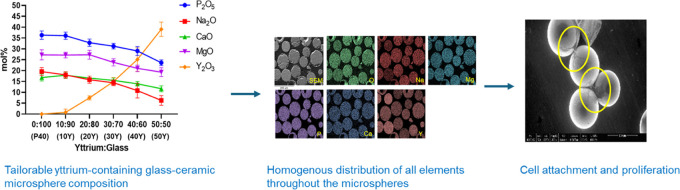

This study presents the development and characterization of high yttrium-content phosphate-based glass-ceramic microspheres for potential applications in bone cancer radiotherapy treatment. The microspheres produced via flame spheroidization, followed by sieving, revealed a lack of aggregation and a narrow size distribution (45–125 μm) achieved across different yttrium oxide to glass ratio samples. Energy dispersive X-ray (EDX) analysis showed a significant increase in yttrium content within the microspheres with increasing yttrium oxide to glass ratio samples, ranging from approximately 1–39 mol % for 10Y–50Y microspheres, respectively. Concurrently, a proportional decrease in the phosphate, calcium, and magnesium content was observed. Further EDX mapping showed a homogeneous distribution of all elements throughout the microspheres, indicating uniform composition. X-ray diffraction profiles confirmed the amorphous nature of the starting P40 glass microspheres, while yttrium-containing microspheres exhibited crystalline peaks corresponding to cubic and hexagonal Y_2_O_3_ and Y(PO_4_) phases, indicating the formation of glass-ceramic materials. Ion release studies revealed the reduction of all ion release rates from yttrium-containing microspheres compared with P40 microspheres. The pH of the surrounding media was also stable at approximately pH 7 over time, highlighting the chemical durability of the microspheres’ produced. In vitro cytocompatibility studies demonstrated that both indirect and direct cell culture methods showed favorable cellular responses. The metabolic and alkaline phosphatase activity assays indicated comparable or enhanced cell responses on yttrium-containing microspheres compared to the initial P40 glass microspheres. Overall, these findings showed that significantly high yttrium-content phosphate glass-ceramic microspheres could be produced as versatile biomaterials offering potential applications for combined bone cancer radiotherapy treatment and bone regeneration.

## Introduction

Internal radiation therapy is a cancer treatment method that involves positioning radioactive sources inside the body, typically near or directly within a tumor.^1^ This treatment can be particularly effective against cancers, especially when the response to chemotherapy is poor or when external beam radiotherapy is not possible due to the location of the cancerous tissue.^2^ Effective treatment requires delivering radiation that maximizes the dose to malignant cancer cells while minimizing exposure to adjacent healthy cells.^3^ One strategy that has successfully been used to deliver internal radiotherapy is the use of radionuclide-doped microspheres.^4^

Various biomaterials have been employed to deliver specific radionuclides that emit α, β, or γ radiation for internal radiation therapy. β-emitting (β) radionuclides are extensively employed owing to their capability to administer high radiation doses with adequate tissue penetration.^5^ The selection of an appropriate radionuclide is guided by its properties, ensuring the optimal delivery of the desired radiation dose over the necessary distance to maximize treatment efficacy for the targeted tissue or organ.^6^

Yttrium 90 (^90^Y) is a radionuclide that has been used to clinically deliver internal radiotherapy. Nonradioactive ^89^Y is activated to the pure β-emitter ^90^Y by neutron bombardment prior to implantation, with the resulting ^90^Y having a half-life of 64.2 h, a tissue penetration depth ranging from 2.5 to 11 mm, and the capability of delivering therapeutic doses of ionizing radiation.^7^ Currently, two types of ^90^Y-containing microspheres are commercially available for use in selective internal radiation therapy (SIRT) (also known as radioembolization) for the treatment of unresectable hepatocellular carcinoma.^8^ SIR-Spheres (Sirtex Medical, Sydney, Australia) are resin-based microspheres comprised of a proprietary biocompatible microsphere coated with a cross-linked cation exchange polystyrene resin.^9^ TheraSphere (Boston Scientific, United Kingdom) are alumina silicate glass microspheres produced via traditional melt-quenching technique involving yttrium oxide (Y_2_O_3_), aluminum oxide, and silicon dioxide, followed by flame spheroidization.^10^

Glass microspheres are highly appealing for internal radiotherapy delivery, as the nonradioactive isotope can be integrated into the glass’s chemical and physical structure during its manufacture.^11^ Once the glass microspheres are manufactured, neutron activation occurs, which ensures an inherent safety benefit by minimizing radiation exposure during their fabrication. Once irradiated, the glass microspheres need to possess high chemical durability in order to prevent leaching of the radionuclide and irradiating the patient away from the target site.^12^ The time from neutron activation of the microspheres to their delivery in the clinic is also used to ensure the administration of specific doses.^13^ Despite this, significant decay of the microsphere radioactivity occurs before treatment has started due to the relatively short half-life of ^90^Y.^14^

The amount of Y_2_O_3_ that can be incorporated into the structure of silicate-based glasses is currently limited to around 18 mol % and requires a high-temperature melting process due to the Y_2_O_3_ melting temperature of 2425 °C.^15^ Several studies in the literature have reported on the use of phosphate-based glasses as versatile vectors for internal radiotherapy due to their ability to encapsulate radionuclides, facilitating targeted radiation therapy while minimizing damage to surrounding healthy tissues.^16−18^ However, in phosphate-based glasses, Y_2_O_3_ addition has been limited to around 5 mol % as further addition resulted in crystallization of the glass, although Martin et al. incorporated approximately 31 mol % Y_2_O_3_ within yttrium alumino-phosphate glasses.^19,20^ To enhance therapeutic efficacy, there is a desire to increase the yttrium content within glass vectors, as the low concentration currently incorporated reduces the maximum dose of radiation, thereby necessitating a longer activation time through neutron bombardment to achieve the desired radiation dose levels.^21^

The biocompatibility and controlled release properties of certain phosphate-based glasses have meant they have also become valuable candidates for bone repair and regeneration applications.^22^ Phosphate-glass microspheres have gathered significant research interest for their dual potential to serve as carriers for internal radiotherapy delivery while simultaneously facilitating bone repair. This dual functionality arises from their ability to encapsulate radionuclides for targeted therapy and act as biocompatible scaffolds, promoting bone regeneration and presenting a promising avenue for multifaceted medical applications.

This study reports on the processing and characterization of high yttrium-content phosphate-based glass-ceramic microspheres for potential applications in delivering internal radiotherapy for the treatment of bone cancers. In vitro cytocompatibility studies have also been performed to assess the microspheres’ ability to support cell growth and proliferation and to facilitate the bone repair and regeneration of damaged tissue following devastation due to bone cancer and its associated treatments.

## Materials and Methodology

### Glass Fabrication

The P40 phosphate glass formulation (40P_2_O_5_·16CaO·24MgO·20Na_2_O mol%) was prepared using the following precursors: sodium dihydrogen phosphate (NaH_2_PO_4_), calcium hydrogen phosphate (CaHPO_4_), calcium carbonate (CaCO_3_), and magnesium hydrogen phosphate trihydrate (MgHPO_4_·3H_2_O) (Sigma Aldrich, UK). The precursors were accurately weighed based on the specified composition and thoroughly mixed before being heated in a 5% Au/Pt crucible at 350 °C for 30 min. This initial heating phase aimed to dehydrate the samples and eliminate CO_2_. Subsequently, the mixture was melted at 1150 °C with a heating rate of 10 °C/min and maintained at this temperature for 90 min. The resulting molten glass was quenched between two stainless steel plates at room temperature.

### Microsphere Manufacture

Microspheres were manufactured according to the method previously described in ref (42). The P40 phosphate glass was ground using a Retsch PM100 milling machine and sieved into a size range of 45–63 μm. For yttrium-containing microspheres, the glass was mixed with the corresponding ratio of yttrium(III) oxide (ACROS Organics, UK) using a Vortex-Genie 2 (Sigma-Aldridge, UK) benchtop vortex for 1 min to achieve homogeneous mixing of the two powders (see Table 1). The P40 glass and the P40/yttrium oxide mixtures were processed into microspheres using a flame-spheroidization method employing an oxy-acetylene thermal spray gun (MK74, Metallisation Ltd., UK). Processed microspheres were washed with deionized water and left to dry in a 50 °C oven overnight. The microspheres were then sieved into a size range of 45–125 μm using laboratory test sieves (Endecotts, UK).

**Table 1:** P40:Yttrium Oxide Ratio and the Corresponding Sample Codes

sample code	ratio of P40 glass to yttrium oxide
P40	100:0
10Y	90:10
20Y	80:20
30Y	70:30
40Y	60:40
50Y	50:50

### Scanning Electron Microscopy

Morphological analysis of the microspheres was conducted utilizing a JSM-6490LV instrument (JEOL, USA). Selected microsphere samples were mounted on carbon tabs affixed to aluminum stubs and sputter-coated with approximately 15 nm of platinum under an argon atmosphere.

To investigate the internal structures, microspheres were embedded in a cold-set epoxy resin. The resin blocks were then polished with SiC paper and a polishing cloth embedded with diamond paste down to a 1 μm finish using industrial methylated spirit (IMS) (Sigma Aldrich, UK) as the lubricant. After polishing, the resin block was placed in an ultrasonic bath with IMS for 5 min and allowed to dry. Finally, the samples were sputter-coated with 15 nm of carbon using a Quorum Q150V (Quorum, UK) for energy-dispersive X-ray (EDX) analysis.

### EDX Spectroscopy

Compositional analysis was conducted on both microspheres mounted on carbon tabs fixed to aluminum stubs and those embedded in the resin. The samples were coated with a layer of carbon using a Q150T turbo-pumped Sputter Carbon Coater (Quorum, UK). For energy-dispersive EDX and mapping, an Oxford Instruments INCA EDX system equipped with a Si-Li crystal detector was integrated with a JSM-6490LV SEM, operating at an accelerating voltage of 15 kV and a working distance of 10 mm.

### Powder X-ray Diffraction (XRD)

To assess the amorphous or crystalline nature of the microspheres, a Bruker D8 Advanced X-ray diffractometer (Bruker-AXS, Karlsruhe, Germany) operating at room temperature with a Ni-filtered Cu Kα radiation source was used. Data were collected at 0.02° intervals across a 10–70° range over a duration of 10 min. The obtained data were then analyzed using DIFFRAC.EVA software (DIFFRAC-plus suite, Bruker-AXS), which facilitated phase identification by referencing the International Centre for Diffraction Data (ICDD) 2021 database.

### Ion Release Studies

The ion release profiles of the microspheres were assessed, according to the method previously described in ref (9), by immersing 400 mg of the microspheres in 40 mL of ultrapure Milli-Q water at 37 °C. At each time interval (3, 7, 14, 21, and 28 days), the dissolution medium was filtered and replaced. The concentrations of sodium, phosphorus, calcium, magnesium, and yttrium ions were quantified using inductively coupled plasma mass spectrometry (ICP-MS, ThermoFisher iCAP-Q model). Additionally, the pH of the solutions was recorded at each time point with an InLab Pure Pro-ISM pH electrode (Mettler Toledo, UK).

### Microsphere Sterilization and Preparation of Conditioned Media

As described by Milborne et al., sterilization of the microspheres was achieved through two successive 10 min washes with 100% ethanol. Following this, the microspheres were allowed to dry completely overnight at room temperature within a sterile Class 2 microbiological safety cabinet to ensure aseptic conditions and to reduce the risk of microbial contamination.

For preparing the conditioned medium with microsphere ion extracts for MG63 cells, 100 mg/mL sterile microspheres were incubated in standard cell culture medium (DMEM supplemented with 10% fetal calf serum, 1% penicillin–streptomycin, 1% l-glutamine, 1% nonessential amino acids, and 1.5% ascorbic acid; ThermoFisher, UK) at 37 °C with 5% CO_2_. The conditioned medium containing the ion extracts was collected and replaced with fresh medium of equal volume every 48 h. Prior to administration to the cells, the solutions were filtered through 0.22 μm syringe filters to eliminate any debris or precipitate.^9^

### In Vitro Indirect Cell Culture Studies

For the indirect culture method using microsphere-conditioned media, the human osteoblast-derived cell line MG63 (obtained from the European collection of authenticated cell cultures—ECACC) was seeded at a density of 10 000 cells/cm^2^ in 300 μL of standard cell culture medium in 48-well plates. After 48 h, cells were washed with PBS and 300 μL of the corresponding conditioned media was added. Control groups included cells cultured with either unconditioned standard medium (+ve) or standard cell culture medium supplemented with 5% DMSO (−ve). Media was refreshed every 48 h. The experiment was conducted with two independent biological replicates, each including three experimental replicates per condition.^9^

### In Vitro Direct Cell Culture Studies

Direct seeding of cells onto the microspheres was performed according to the method previously described in ref (9). Low-adherent 48-well plates (Sigma Aldrich, UK) were coated with a 1% (w/v) solution of poly(2-hydroxyethyl methacrylate) (poly-HEMA, Sigma Aldrich, UK). This was accomplished by dissolving powdered poly-HEMA in preheated 65 °C absolute ethanol, applying the solution to the well plates, and incubating them overnight at 37 °C to allow for ethanol evaporation. The plates were washed three times with PBS. Ten milligrams of sterilized microspheres from each formulation was added to the wells, and MG63 cells were seeded at a density of 10 000 cells/cm^2^. Each well received 300 μL of a standard cell culture medium. Cells were cultured for 7 days at 37 °C and 5% CO_2_, with media changes occurring every 48 h.

### Cell Metabolic Activity

The metabolic activity of MG63 cells was assessed on days 2 and 7 using the Alamar Blue assay. To each well, 300 μL of Alamar Blue solution (prepared as a 1:9 mixture of Alamar Blue and Hank’s Balanced Salt Solution) was added and incubated for 90 min at 37 °C with 5% CO_2_, followed by an additional 10 min of shaking at 150 rpm. From each condition, three 100 μL aliquots were transferred to a 96-well plate. Fluorescence measurements were conducted using an FLx800 fluorescence microplate reader (BioTek Instruments Inc.) with excitation at 530 nm and emission at 590 nm.^45^

### Alkaline Phosphatase Activity

On day 7, the cells were washed three times with warm (37 °C) PBS and then immersed in 1 mL of deionized water. The samples underwent three freeze–thaw cycles to lyse the cells and release their nuclear content. Alkaline phosphatase (ALP) activity in MG63 cells was quantified using a Granutest 25 ALP assay (Randox, UK). Three 50 μL aliquots of the cell lysate were placed into a 96-well plate, each followed by 50 μL of ALP substrate (p-nitrophenyl phosphate at 10 mM in a diethanolamine buffer at 1 mM, pH 9.8, with 0.5 mM MgCl_2_). The plates were gently shaken for 5 min, and absorbance was measured at 405 nm with an FL×800 microplate colorimeter (BioTek Instruments) every 5 min for 45 min until the readings stabilized. This procedure was applied to cells grown under both indirect and direct culture conditions.

### DNA Content Assay

Cell lysates used for ALP activity measurement were thoroughly mixed for 30–60 s using a vortex mixer, and 100 μL of each sample was transferred into a 96-well plate. Hoechst 33258 stain was prepared by dissolving 1 mg of bis(benzimide) in 1 mL of double-distilled water and then diluting the solution to a 1:50 ratio in TNE buffer. DNA standards were created using calf thymus DNA (Sigma, UK) and TNE buffer (10 mM Tris, 2 M NaCl, and 1 mM EDTA in deionized water, adjusted to pH 7.4) to establish a standard curve for DNA concentration. To each well, 100 μL of Hoechst 33258 stain was added and mixed on a plate shaker for 5 min at 150 rpm. The fluorescence was measured using an FLx800 plate reader (BioTek Instruments) with an excitation wavelength of 360 nm and an emission wavelength of 460 nm.

### Statistical Analysis

Two independent experiments were performed, and results are presented as the mean ± standard error of the mean unless specified otherwise. Statistical analysis was performed using the Prism software package (version 9.2.0, GraphPad Software, San Diego, CA, www.graphpad.com). A two-way analysis of variance was performed, followed by a Tukey’s multiple comparison test. The mean difference was deemed statistically significant at a threshold of 0.05 with a 95% confidence interval.

### Cell Imaging

For imaging of cell attachment to microspheres, at day 7, the MG63s were washed three times with warm (37 °C) PBS and fixed with 4% paraformaldehyde for 10 min. Fixative was then removed, and the sample was washed twice in deionized water. For environmental scanning electron microscopy (ESEM), after fixation, microspheres and cells were carefully isolated using a glass pipet and mounted onto the stage of the FEI Quanta 650 ESEM microscope for analysis.

## Results

### Morphological Analysis

**Figure 1:**
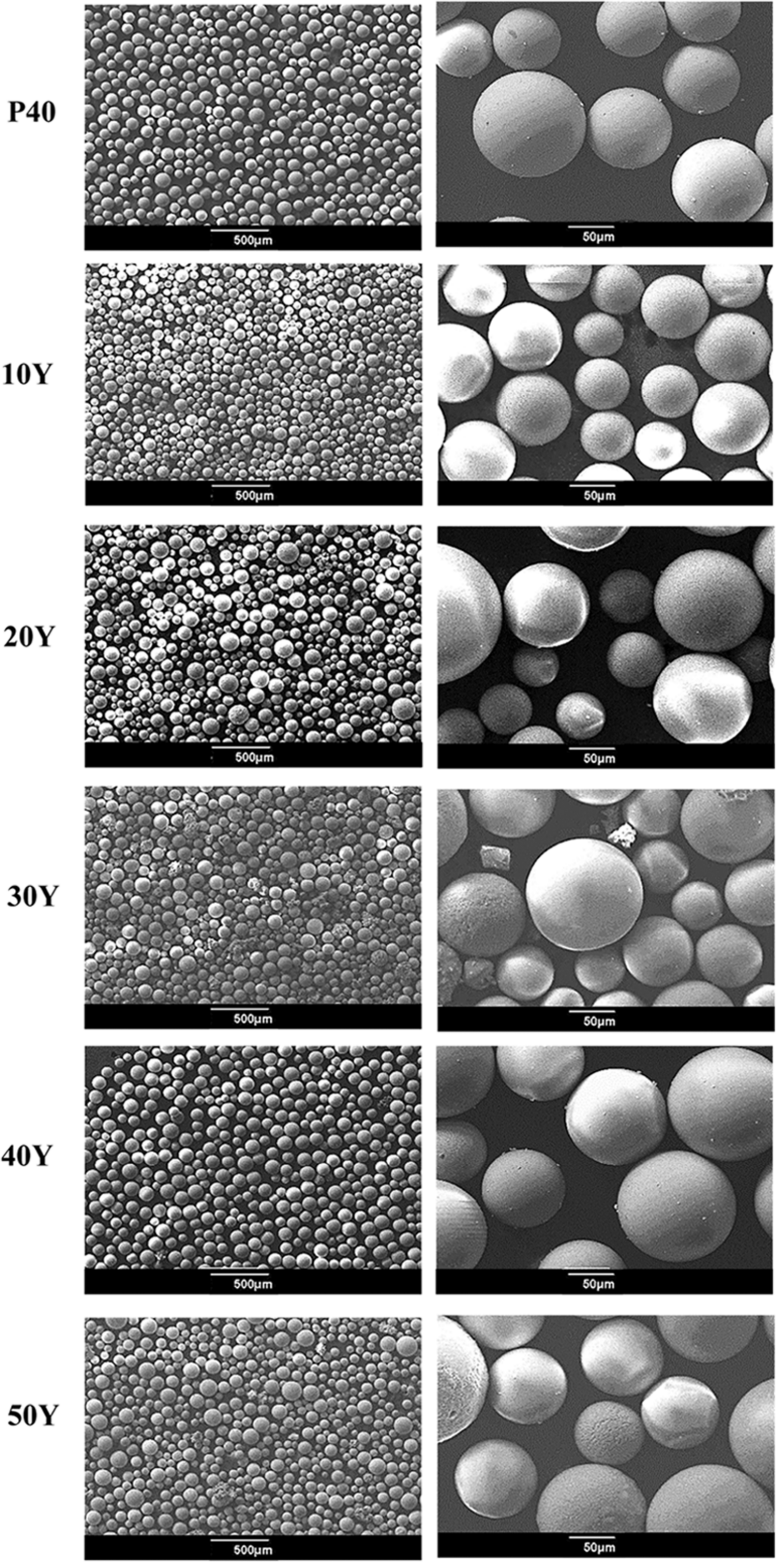
SEM images depicting the morphology of the yttrium-containing microspheres produced.

SEM analysis revealed that after processing the glass particles via flame spheroidization and sieving within a 45–125 μm size range, a high yield of spherical microspheres was produced. As seen in Figure 1, a lack of aggregation and narrow size distribution was achieved using this process for each of the yttrium oxide to glass ratio samples prepared prior to spheroidization.

### Compositional Analysis

**Figure 2:**
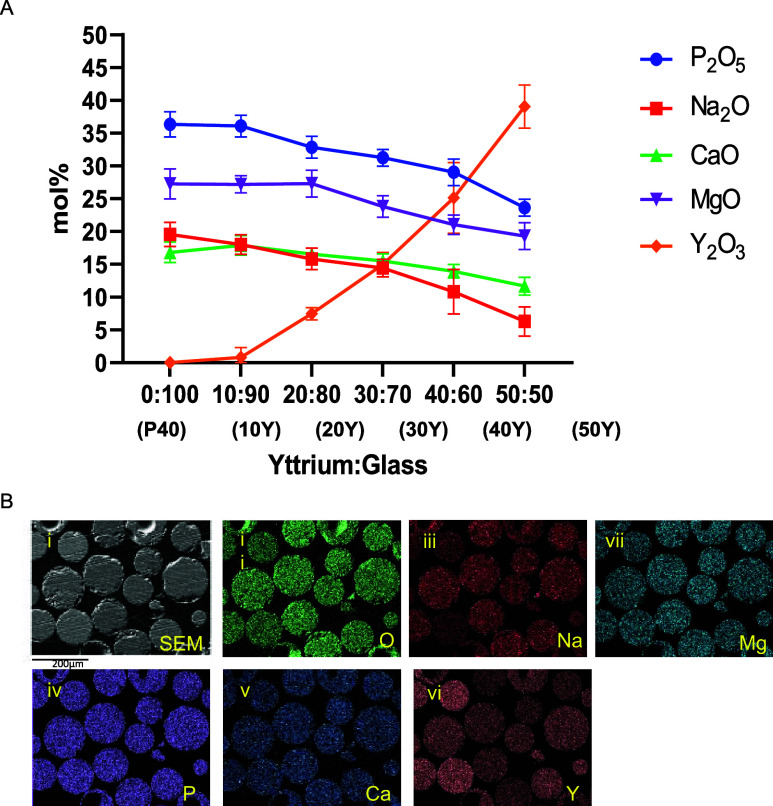
(A) Elemental composition determined EDX of the yttrium-containing microspheres produced via flame spheroidization. Results are presented as the mean ± standard error of the mean (*n* = 10). (B) EDX mapping of resin-embedded 30Y microspheres showing the homogeneous distribution of all of the elements throughout the microspheres produced the (i) SEM image of resin-embedded and polished samples, (ii) Oxygen, (iii) sodium, (iv) magnesium, (v) phosphorus, (vi) calcium, and (vii) yttrium content are observed.

EDX analysis was performed to confirm the chemical composition of the microspheres manufactured at each yttrium oxide/glass ratio explored. As the amount of yttrium oxide mixed with the glass prior to processing increased, a subsequent significant increase in the yttrium content within the microspheres was observed. The yttrium oxide content increased from ∼0.8, 7.5, 15.0, 25.1, and 39.1 mol % for the 10Y, 20Y, 30Y, 40Y, and 50Y microspheres, respectively. However, a proportional decrease in all of the other glass elements was also observed (see Figure 2A). Phosphate content decreased from ∼36 mol % in P40 and 10Y microspheres to 23.6 mol % within 50Y microspheres. Calcium oxide content decreased from 16.8 mol % in P40 microspheres to 19.6, 16.6, 15.5, 14.0, and 11.7 mol % for the 10Y, 20Y, 30Y, 40Y, and 50Y microspheres, respectively. Similarly, magnesium oxide content decreased incrementally when moving through the series from 10Y to 50Y. Magnesium oxide content in 50Y microspheres of 19.3 mol % was approximately two-thirds of the content of the magnesium oxide content in comparison to P40 microspheres (27.3 mol %).

To explore the distribution of the elements within the microspheres produced, EDX mapping (of representative resin-embedded microspheres, which were then subsequently polished to reveal the inner structure) of P40, 30Y, and 50Y microspheres was performed. For P40 microspheres, all of the glass-forming elements (O, N, Mg, P, Ca) were distributed evenly throughout the microspheres. The yttrium-containing microspheres had the same elements detected as well as yttrium being homogenously distributed throughout the whole body of the microspheres (see Figure 2B).

### XRD Analysis

**Figure 3:**
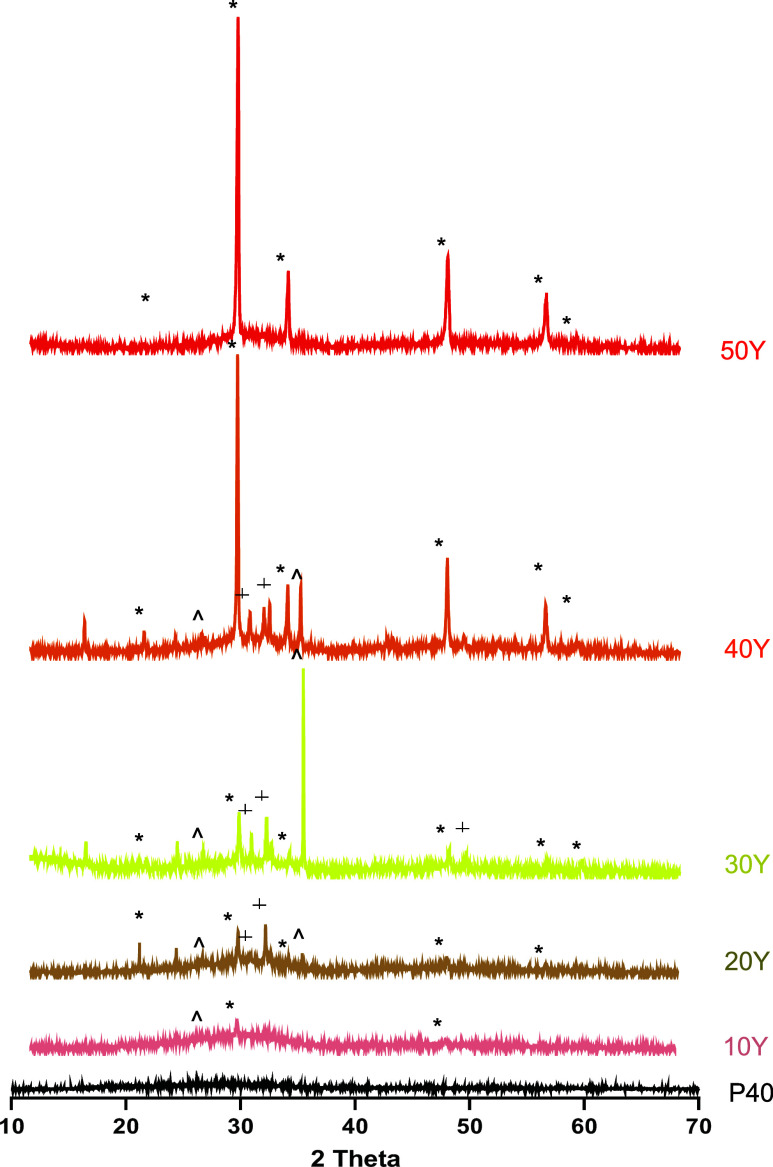
XRD spectra of P40 SMS (black), 10Y (pink), 20Y (brown), 30Y (gold), 40Y (orange), and 50Y (red) solid microspheres. The crystalline peaks matched for cubic Y_2_O_3_ (*) (01-079-1257), hexagonal Y_2_O_3_ (+) (01-076-7397), and Y(PO4) (∧)(01-084-0335).

Figure 3 shows the XRD profiles for the solid microspheres made from the P40 parent glass and at each yttrium:glass ratio. A single broad halo peak at 2θ values of ∼30–32° was observed for the P40 solid microspheres, and the absence of any detectable crystalline peaks confirmed the amorphous nature of the glass microspheres produced. The profiles for the yttrium-containing microspheres post-processing revealed the presence of sharp crystalline peaks. For the 10Y sample, peaks at ∼29 and 49° were observed, which were matched to cubic Y_2_O_3_ according to powder diffraction file 01-079-1257 (ICDD database). An additional peak at ∼26° was also seen, which corresponded to Y(PO_4_) (ICDD 01-084-0335). For the 20Y microspheres, sharp peaks at ∼29, 34, 49, and 58° were observed, which were matched to cubic Y_2_O_3_ (01-079-1257 ICDD) as well as peaks at ∼26 and 35°, which corresponded to Y(PO_4_) (ICDD 01-084-0335). Additional peaks were also seen at ∼30 and 32°, which were matched to hexagonal Y_2_O_3_ according to powder diffraction file 01-076-7397 (ICDD database). Both 30Y and 40Y microspheres showed the same peaks corresponding to cubic Y_2_O_3_ (01-079-1257 ICDD), Y(PO_4_) (ICDD 01-084-0335), and also hexagonal Y_2_O_3_ (01-076-7397 ICDD). The 50Y microsphere profile showed peaks only at ∼29, 34, 49, 58, and 61° 2θ values, which corresponded to cubic Y_2_O_3_ (according to the file 01-079-1257 ICDD database).

From here on, only the 30Y and 50Y microspheres were chosen for further characterization and study. The 30Y was selected due to its yttrium content (∼15.0 mol % ± 1.8) being comparable to that of TheraSphere and containing a P_2_O_5_ content of ∼30 mol %. While 50Y microspheres were selected as these had the highest yttrium content (39.1 mol % ± 3.3).

### Ion Release Studies

Ion release studies were performed to assess the durability of the yttrium-containing microspheres compared to that of P40 microspheres and the effect of yttrium addition on ion release kinetics.

**Figure 4:**
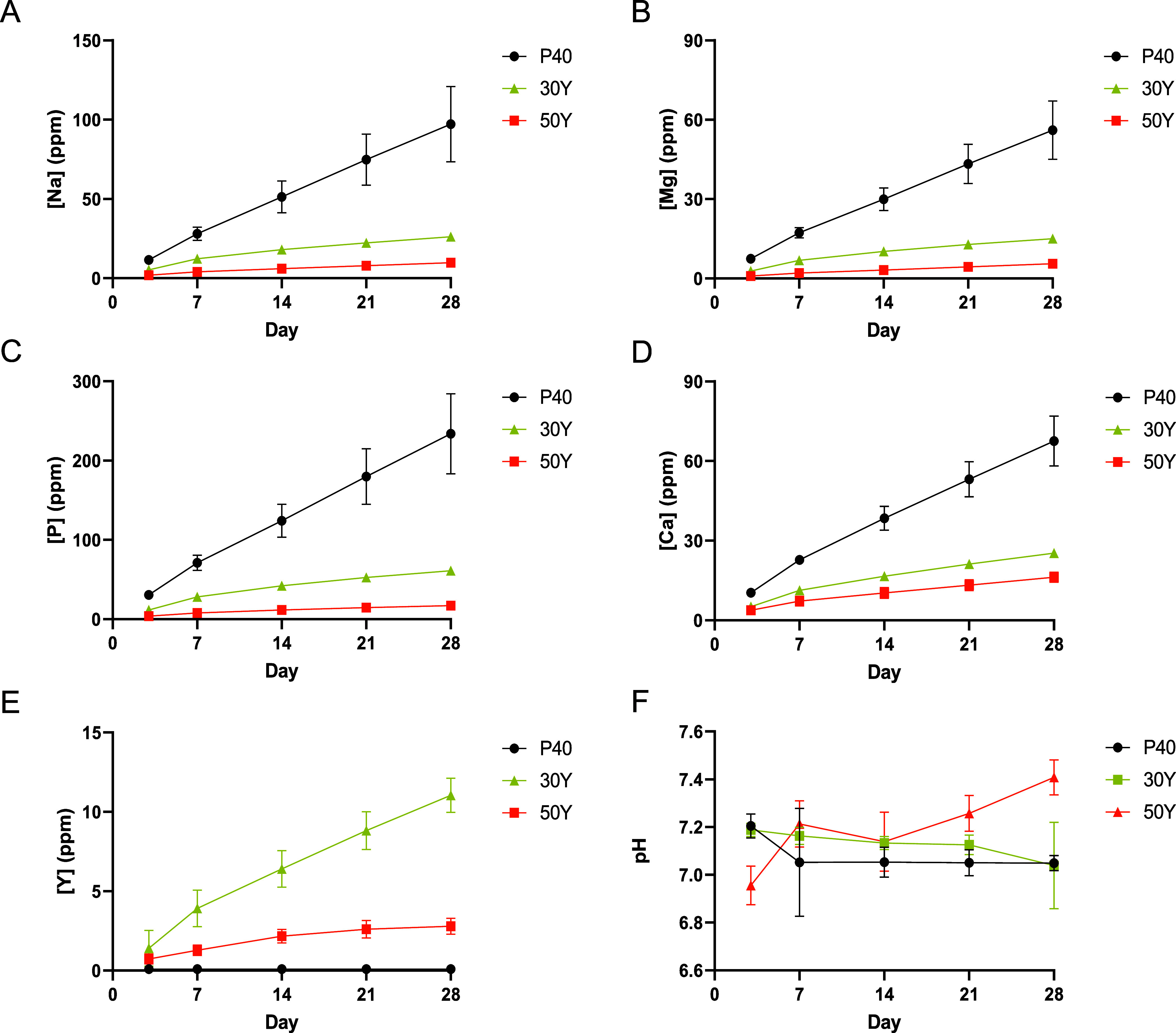
Cumulative ion release profile of (A) [Na], (B) [Mg], (C) [P], (D) [Ca], and (E) [Y] measured via ICP-MS of solid P40, 30Y, and 50Y microspheres immersed in Milli-Q water over a 28-day period. (F) pH of Milli-Q water during 28 days of P40, 30Y, and 50Y solid microsphere immersion within the solution. (Standard deviation error bars are also included in the data; three independent repeats were performed for studies at each time for each microsphere composition).

Figure 4 shows the cumulative ion release profiles for P40, 30Y, and 50Y solid microspheres calculated from measurements obtained via ICP analysis over 28 days. The ions released from each formulation exhibited a linear relationship with time and were released consistently. The P40 solid microspheres degraded much faster than the 30Y and 50Y microspheres, revealing ion release rates that were statistically significant and higher in comparison to the yttrium-containing microspheres (i.e., Na and P: *p* < 0.0001; Mg and Ca: vs 30Y *p* < 0.01, vs 50Y *p <* 0.001). As the yttrium content in the microspheres increased, a decrease in the ion release rates was also observed.

The P40 microspheres released Na^+^ (∼3.5 ppm/day), Mg^2+^ (2.0 ppm/day), and P (8.4 ppm/day) at an approximate rate of around 3.5 times greater than that of the 30Y microspheres and around 10 times higher rate compared to 50Y microspheres. P was the only ion that was released at a statistically significantly higher release rate (2.2 ppm/day) from 30Y microspheres in comparison to 50Y microspheres (0.6 ppm/day) (*p* < 0.01). The yttrium-containing microspheres released only very small amounts of yttrium ions in comparison to all of the other elements. The 30Y microspheres revealed the highest release rate of Y^3+^ ions (at ∼0.4 ppm/day), whereas the 50Y microspheres, which contained a greater amount of yttrium, revealed an approximate release rate of 0.1 ppm/day.

Figure 4F illustrates the pH changes in Milli-Q water during the 28-day immersion of the microspheres. For the solution with P40 solid microspheres, the pH dropped from ∼7.2 on day 3 to ∼7.0 by day 7 and then remained stable for the remainder of the study. 30Y solution had a comparable value to the P40 sample at both day 3 and day 28, albeit with a slight decrease in pH which occurred more gradually. A pH of ∼6.9 for 50Y was observed at day 3, which then increased to ∼7.4 by the end of the 28-day period.

### Indirect In Vitro Cell Culture Studies

**Figure 5:**
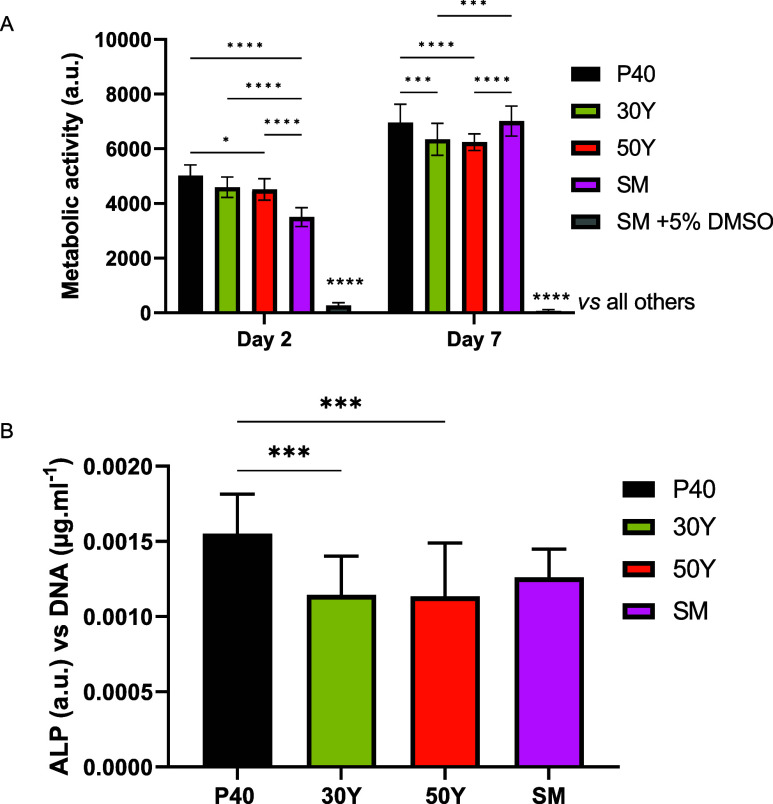
(A) Evaluation of cell metabolic activity in the indirect culture of P40, 30Y, and 50Y solid microspheres at days 2 and day 7. ****p < 0.0001, ***p < 0.001, *p < 0.05. (B) Evaluation of ALP activity in the indirect culture of P40, 30Y, and 50Y solid microspheres at day 7. ***p < 0.001. Results are presented as mean ± standard error of the mean. Two independent experiments were performed, each with *n* = 3 replicate wells.

To assess the cytocompatibility of the produced microspheres, an indirect cell culture approach was used. This method involved treating osteoblast-like MG63 cells with media conditioned by the microspheres to analyze their biological responses to the dissolution products released over time. Standard medium (SM) and SM with 5% DMSO served as positive and negative controls, respectively. Analysis of metabolic activity via the Alamar Blue assay showed a significant increase in cell response when both 30Y and 50Y microsphere-conditioned media were added between day 2 and day 7 (D2 vs D7: *p* < 0.0001). This increase in metabolic activity was also seen for cells treated with SM, but cells where SM + 5% DMSO was applied revealed no apparent increase in metabolic activity from day 2 to day 7 (D2 vs D7: *p* < 0.0001). At day 2, cells treated with P40, 30Y, and 50Y conditioned media had a significantly greater metabolic response compared to those treated with SM and SM + 5% DMSO (*p* < 0.0001). No statistically significant difference was observed between cells treated with 30Y and 50Y conditioned media (*p* > 0.05). There was also no statistically significant difference between the cells treated with 30Y and 50Y conditioned media at day 7 (*p* > 0.05). At day 7, cells treated with P40 and SM media revealed a statistically significant increased metabolic response compared to the yttrium formulations (*p* < 0.001); however, no statistically significant differences were detected between P40 and SM (*p* > 0.05) (see Figure 5A).

ALP activity was also measured as an early marker of osteogenic differentiation in MG63 cells after 7 days of indirect culture from P40, 30Y, and 50Y microsphere formulations and SM. The ALP activity was normalized to the DNA content of the cells under investigation. At day 7, there was no statistically significant difference in ALP activity between cells grown in SM and both 30Y and 50Y conditioned media (*p* > 0.05). Cells grown in P40 media had statistically significantly higher ALP activity compared to cells grown in the two yttrium-containing microsphere media (vs 30Y and 50Y: *p* < 0.001) (see Figure 5B).

### Direct In Vitro Cell Culture Studies

**Figure 6:**
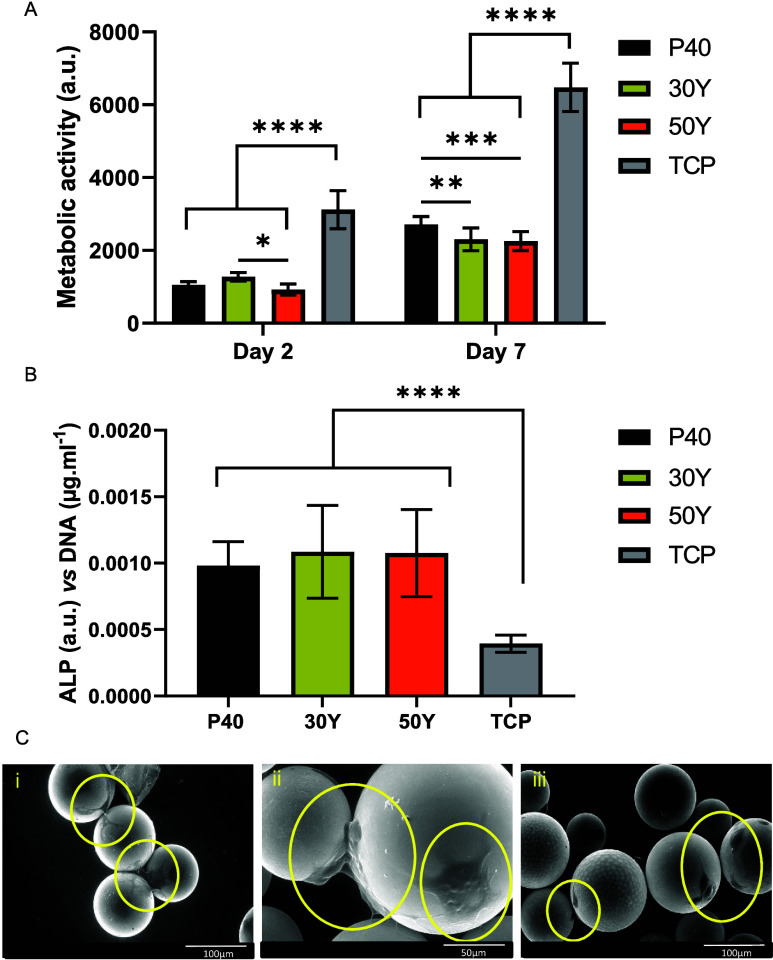
(A) Evaluation of cell metabolic activity in the direct culture of P40, 30Y, and 50Y solid microspheres at days 2 and day 7. ****p < 0.0001, ***p < 0.001, **p < 0.01, *p < 0.5. Results are presented as mean ± standard error of the mean. Two independent experiments were performed, each with *n* = 3 replicate wells. (B) Evaluation of ALP activity in the direct culture of P40, 30Y, and 50Y solid microspheres at day 7. ***p < 0.001. Results are presented as mean ± standard error of the mean. Two independent experiments were performed, each with *n* = 3 replicate wells. (C) SEM images of (i) P40, (ii) 30Y, and (iii) 50Y solid microspheres after 7 days of direct culture with MG63 cells. The yellow circles highlight areas where cells have attached and formed colonies on the surface of the microspheres.

MG63 cells were also directly seeded onto P40, 30Y, and 50Y solid microspheres to assess the effect of direct physical contact on cellular responses and the microspheres’ ability to provide a suitable surface to facilitate cell growth and proliferation. Analysis of metabolic activity at day 2 revealed that there was no statistically significant difference in metabolic activity between cells grown on P40 microspheres and the two yttrium-containing microsphere formulations (*p* > 0.05). Cells cultured on 30Y microspheres revealed higher metabolic activity at day 2 compared to those grown on 50Y (*p* < 0.05). Cells grown on any of the three microsphere formulations displayed statistically significantly lower metabolic activity compared to TCP control (*p* < 0.0001) (see Figure 6A).

Also, a statistically significantly higher metabolic activity was seen in cells cultured on each of the three microsphere formulations and TCP at day 7 in comparison to day 2 (*p* < 0.0001). At day 7, no statistically significant difference in metabolic activity between the cells cultured on 30Y and 50Y microspheres (*p* > 0.05) was observed. However, metabolic activity was statistically significantly lower than those cultured on P40 microspheres (vs 30Y: p < 0.01; vs 50Y: p < 0.001) (see Figure 6A).

After 7 days, the ALP activity of MG63s grown directly on the microspheres and TCP was determined and normalized to the DNA concentration. Statistically significantly higher ALP activity was recorded for cells grown on P40, 30Y, and 50Y microspheres in comparison to those grown on the TCP control (*p* < 0.0001). There was no statistically significant difference in ALP activity detected between cells grown on P40 and the two yttrium-containing microsphere formulations (*p* > 0.05) (see Figure 6B).

MG63 cells directly cultured onto the microspheres were visualized using ESEM at day 7. Cells were seen adhered onto the P40, 30Y, and 50Y microsphere surfaces and appeared to be displaying lamellipodia and filopodia projections, which bridged adjacent neighboring microspheres and were spread over the microsphere surfaces (see Figure 6C).

## Discussion

In this work, a novel processing method was developed to significantly increase the level of yttrium content that could be incorporated into phosphate-based glass.^23^ A study by Arafat et al. investigating the crystallization behavior of glasses within the system 45P_2_O_5_—(30 – *x*) Na_2_O–25CaO–*x*Y_2_O_3_—(where *x* = 0–10) found that the addition of Y_2_O_3_ was limited to ∼5 mol % when attempting to prepare the formulations via conventional glass melt processes, as further addition resulted in crystallization of the glass.^22^ This demonstrated the difficulties often encountered when trying to incorporate large quantities of Y_2_O_3_ within a phosphate-based glass. The current methodology developed allowed for the yttrium content to be varied by simply altering the Y_2_O_3_ to glass particle ratios prior to the spheroidization process. This resulted in the production of uniform solid microspheres containing varying yttrium levels (ranging from ∼1 to 39 mol %). This method was capable of producing microspheres that had equivalent and enhanced yttrium content in comparison to clinically available aluminosilicate glass microspheres used for internal radiotherapy applications (Therasphere). The 30Y microspheres were chosen for further characterization and study due to their yttrium content (15 mol % ± 1.8) being comparable to that of Therasphere while also containing a P_2_O_5_ content of ∼30 mol %. The 50Y microspheres were studied further as these had the highest yttrium content (39 mol % ± 3.3), and it was postulated that they might retain some of the beneficial features from their parent P40 glass, such as the release of therapeutic ions (such as Ca, Mg, and Na). The authors believe that the yttrium oxide content achieved within the 50Y microsphere composition is the highest achieved within a phosphate glass matrix to produce uniform microspheres.

In addition to elevated yttrium content, microspheres offer enhanced delivery characteristics compared to irregularly shaped particles and can be effectively administered through minimally invasive surgical injection techniques.^24^ This is important for internal radiotherapy applications, where the ability to administer them easily and accurately is vital in order to maximize their therapeutic efficacy.^25^

EDX analysis confirmed that the addition of Y_2_O_3_ resulted in the formation of microspheres that had a reduced content of all elements present in the parent P40 glass formulation after processing. With increasing Y_2_O_3_ addition, proportional decreases in these elements were observed (Figure 2A). EDX mapping of resin-embedded 30Y and 50Y microspheres was performed to establish whether any of the elements were concentrated at regions within the microspheres. The mapping showed that all of the elements were homogeneously distributed throughout the whole of the yttrium-containing microspheres and did not appear to be concentrated at the surface (see Figure 2B). Tesfay et al. produced Y-doped bioactive glass spherical powders based on 58S (60 mol % SiO_2_, 35 mol % CaO, and 5 mol % P_2_O_5_) using a spray pyrolysis method. They also achieved local Y distribution dispersed homogeneously throughout their particles. However, the Y content was only 7–11 mol %, and particles were less than 1 μm in size.^26^ Ghahramani et al. obtained yttrium aluminum silicate microspheres, around 20–50 μm in size, using a sol–gel method via an aqueous solution of Y(NO_3_)_3_ and Al(NO_3_)_3_ being added to tetraethyl orthosilicate (TEOS) and pumped into stirred silicone oil. In their study, SEM and EDX analyses revealed that the microspheres consisted of two parts: the crust and the core. Y and Al were shown to be distributed in the core, whereas Si was distributed in the crust, which appeared at the periphery of the microspheres.^27^ Sol–gel methods are time-consuming and labor-intensive due to their multistep nature, with a lengthy methodology required to remove residual contaminants. In the present study, a novel single-stage flame spheroidization method was used to rapidly produce high yttrium-containing phosphate-based microspheres with homogeneous elemental distribution. Homogenous size distribution and desired size range can be achieved using laboratory test sieves in specific and appropriate size ranges.

It is proposed that a high yttrium content in the microspheres would be desirable for radiotherapy applications as it may enable more radiation to be delivered per dose of microspheres, leading to the use of fewer microspheres.^28^ A higher yttrium content may also result in shorter neutron activation times and aid with logistical issues involving the time and transportation of the microspheres from the nuclear activation facility to the clinic. Nuclear decay occurs during this period, and the greater the amount of radioactivity, the longer the transit time available in order for the patient to still receive an efficacious radiation dose.^29^ Future research involving neutron activation studies is required to empirically validate that yttrium-containing microspheres can generate therapeutically relevant doses of irradiation. Quantification of the actual radiation dose emitted by the activated ^90^Y within the microspheres would allow the identification and design of the optimal formulation to maximize therapeutic efficacy with minimal adverse effects.

The XRD profiles for the yttrium-containing microspheres produced revealed the presence of crystalline peaks, indicating that the microspheres produced were glass-ceramic in nature and not amorphous samples (see Figure 3). The 10Y microsphere samples had peaks that corresponded to cubic Y_2_O_3_ (ICDD 01-079-1257) and Y(PO_4_) (ICDD No. 01-084-0335). Peaks that corresponded to these two phases were present in 20, 30, and 40Y microspheres, as well as additional peaks that corresponded to the hexagonal phase of Y_2_O_3_ (ICDD 01-076-7397). The 50Y microspheres, which contained the highest yttrium content, revealed only peaks corresponding to the cubic phase of Y_2_O_3_ (ICDD 01-079-1257). Similar results were also seen in a study by Kawashita et al., where ceramic microspheres were formed from solely Y_2_O_3_ or YPO_4_ powder using a high-frequency induction thermal plasma melting technique at flame temperatures estimated between 12 000 and 13 000 °C.^30^ Only peaks that corresponded to cubic Y_2_O_3_ were detected in the Y_2_O_3_ microspheres, whereas weak diffraction peaks corresponding to both cubic and monoclinic Y_2_O_3_ were identified in addition to YPO_4_ peaks in the YPO_4_-derived microspheres. For the YPO_4_ microspheres, it was found that the intensity of the YPO_4_ peaks decreased, while the Y_2_O_3_ peaks increased with increasing plasma flame power. This was attributed to the loss of P_2_O_5_ due to volatilization. Similarly, the decreased P_2_O_5_ content within 50Y microspheres compared to 40Y, 30Y, and 20Y may have prevented the formation of a YPO_4_ phase.

The addition of yttrium to phosphate glass microspheres not only facilitated their potential use as a vector for radiotherapy delivery but also significantly improved the durability of phosphate glasses.^31^ Other transition oxides such as TiO_2_, Al_2_O_3_, and Fe_2_O_3_ have been incorporated into phosphate glasses and have been shown to improve some of the physical properties, such as their rapid degradation within aqueous media that can limit their clinical applications.^32−34^ The addition of certain transition oxides has been shown to decrease degradation rates within phosphate-based glasses by up to 3–4 orders of magnitude.^35^ Classical molecular dynamics simulations conducted on the structure of yttrium-doped phosphate-based glasses showed that yttrium oxide up to 6 mol% acted as a network modifier and resulted in depolymerization of the phosphate network within quaternary phosphate glasses.^36^

A study by Arafat et al. investigated the role of yttrium in phosphate-based glasses in the system 45(P_2_O_5_)–25(CaO)–(30 – *x*)(Na_2_O)–*x*(Y_2_O_3_) mol % (0 ≤ *x* ≤ 5) prepared via melt quenching.^20^ Depolymerization of the glass network was seen with increasing yttrium oxide addition. ^31^P NMR analysis showed an increase of Q^1^ species, from 23 to 42%, which was accompanied by a corresponding decrease of Q^2^ species from 77 to 58% as Y_2_O_3_ addition increased from 0 to 5%. The increasing Y_2_O_3_ content also resulted in a decrease in the phosphate chain length, which was further evidence of depolymerization and the dissociation of metaphosphate chains. As a network modifier, Y^3+^ can occupy the interstitial space between PO_4_ tetrahedra and bond with phosphate glass terminal oxygens, causing a decrease in bridging oxygens (BO) and an increase in nonbridging oxygens (NBO). Previous studies have concluded that yttrium perturbs the glass network strongly by stabilizing the formation of negatively charged species such as nonbridging oxygen atoms.^37^ As it is a high-field strength trivalent cation, yttrium forms strong cross-linking Y–O–P bonds between phosphate chains. Yttrium’s field strength (∼0.60 *e* Å^–2^) is significantly higher than that of magnesium (∼0.46 *e* Å^–2^), calcium (∼0.33 *e* Å^–2^), and sodium (∼0.19 *e* Å^–2^), and this leads to the formation of stronger bonds within the phosphate glass network and explains why an increase is chemical durability was seen with the increasing Y_2_O_3_ content.^21,38^

Previous studies showed that the increase in cross-linking of the phosphate network with the addition of Y_2_O_3_ causes a decrease in the degradation of the glasses since Y–O–P bonds are more resistant to hydration attack than P–O–P bonds.^39^ Classical molecular dynamics simulations showed that when yttrium was incorporated into a ternary phosphate glass series, it bonded to a greater number of phosphate chains (4.2–4.3) in comparison to both calcium (3.8) and sodium (3.1–3.2),^36^ leading to strengthening of the glass against dissolution. Decreased degradation results in lower ion release profiles, which would be beneficial when developing glasses for radiotherapy applications.^40^ The glass needs to be durable while the yttrium is radioactive to avoid leaching of the radionuclide and irradiating the patient away from the target site. In the aforementioned studies on yttrium incorporation within phosphate-based glasses, the Y_2_O_3_ content remained relatively low, less than 6%, due to crystallization of the glass at higher Y_2_O_3_ content.

However, the yttrium content in 30Y and 50Y microspheres of ∼15 and 39 mol % was significantly higher than anything obtained and studied previously in phosphate glasses. Extensive depolymerization of the network occurred, and crystalline phases were present throughout the bulk of the microspheres. The presence of crystalline phases in the yttrium-containing glass-ceramic microspheres likely plays a significant role in the observed slower ion release rates compared with the fully amorphous P40 microspheres. Compared to the more disordered structure of an amorphous glass, the organized crystal lattice presents a greater obstacle for ion movement within the material, and the crystalline phases, such as YPO_4_, can act as diffusion barriers for yttrium ions. This hinders the release of ions from the yttrium-containing glass-ceramic microspheres into the surrounding solution.

Ion release studies demonstrated that an increased yttrium content resulted in a reduction in the release of all ions present in the microspheres. P40 microspheres released phosphorus, calcium, magnesium, and sodium at significantly greater rates in comparison to those observed from 30Y microspheres. Furthermore, the release rate of all of these elements was significantly greater from 30Y microspheres in comparison to those observed from 50Y microspheres. Yttrium was released at the lowest rate from 30Y and 50Y microspheres in comparison to all of the other elements. Despite 50Y microspheres containing more than twice the yttrium content in 30Y microspheres, the 30Y microspheres released approximately 4 times the amount of yttrium ions over 28-day immersion (Figure 4). However, at an approximate release rate of 0.4 and 0.1 ppm/day for 30Y and 50Y microspheres, respectively, the overall content released remained extremely low. Depending on the specific crystalline phases formed, yttrium might be incorporated into more chemically stable environments compared to the amorphous glass network. This can reduce the tendency of yttrium ions to leach out during dissolution. The high durability of yttrium-containing microspheres (30Y and 50Y) is beneficial to prevent yttrium leaching. This durability also ensures localized and sustained radiotherapeutic effects, minimizing irradiation of nontarget tissues and supporting long-term treatment efficacy.

It is expected in physiological or buffered media, such as simulated body fluid (SBF), that even slower ion release profiles would be observed. The complex interplay of pH, ionic strength, ion interactions, and biomolecules within these solutions collectively can affect the dissolution kinetics. Surface layers may form on the glass or glass-ceramic surface, leading to reduced ion release rates.^41^ The yttrium-containing microspheres must have high chemical durability and resistance to degradation within bodily fluids to be used for internal radiotherapy delivery.^42^ The half-life of ^90^Y shows that radioactivity decays to a negligible level within 21 days after neutron bombardment, as such stability and minimal leaching of active radioisotopes during this period would be essential.^12^

Cell culture studies were conducted to evaluate the suitability of the yttrium-containing phosphate microspheres for bone regeneration applications, aiming to determine their cytocompatibility and osteoconductivity. These properties are critical for fostering favorable interactions with host cells and promoting enhanced bone tissue regeneration. Phosphate-based microspheres that possess the dual capability of enhancing bone regeneration while also serving as carriers for radiotherapy offer a distinct advantage over their counterparts limited solely to radiotherapy delivery. They provide a holistic therapeutic approach, addressing both cancer treatment and bone repair in a synergistic manner.

The cytocompatibility results showed that MG63 cells cultured with P40 microsphere-conditioned media demonstrated the greatest levels of metabolic activity after 7 days and were comparable to those of cells cultured in standard media (SM). Cells cultured in both 30Y and 50Y media had significantly lower metabolic activity (*p* < 0.001) than those cultured in SM and P40 media at day 7. However, their relatively high activity and the significant increase in metabolic activity seen from day 2 to day 7 demonstrated their cytocompatibility. Previous in vitro studies of porous glass microspheres of the P40 formulation (40P_2_O_5_·16CaO·24MgO·20Na_2_O mol %) showed that they were biocompatible according to the standard ISO 10993-5. Hossain et al. showed that the proliferation rate of hMSCs grown using an indirect culture method was shown to be greater than 70% of the cells grown in the SM when using an MTT assay.^42,43^

As seen in Figure 4, P40 solid microspheres degraded faster than both the 30Y and 50Y microspheres and hence released a significantly greater amount of the glass-forming ions. The simultaneous exposure and quantity of ions released from the different microsphere formulations to the cells resulted in differences in the cell response. Phosphate, calcium, magnesium, and sodium ions have all been shown to play vital roles in bone metabolism and homeostasis.^44^ These ions are essential for various cellular functions, including signaling, energy production, and maintenance of cellular homeostasis. Extracellular Ca^2+^ plays a key role in the regulation of osteoblastic proliferation and differentiation by influencing the expression of specific Ca^2+^-channel isoforms on osteoblasts.^45^ Enhanced cell proliferation is crucial for the initial phase of bone regeneration, where a robust population of osteoprogenitor cells is required to initiate the repair process. The increased release of ions and subsequent exposure to cells from P40 microspheres was therefore likely responsible for the increased metabolic activity observed at days 2 and 7 when compared to the 30Y and 50Y formulations.

ALP is constitutively active at low levels in all cells, but during the early stages of osteogenic differentiation, its activity significantly increases.^46^ It is therefore used as a marker for the detection of early osteogenic differentiation. Cells exposed to P40 ion release products had significantly greater ALP activity compared to 30Y and 50Y formulations. Similar ALP activity within cells exposed to 30Y and 50Y correlated with the ion release data in that the levels were significantly lower for the two formulations and resulted in no significant difference between them.

When cells were seeded directly onto the microspheres, both the 30Y and 50Y and parent P40 glass microspheres promoted cell growth, as seen by an increase in metabolic activity from day 2 to day 7. Cells cultured on 30Y microspheres showed an initially higher cell metabolic activity at day 2. However, no statistically significant difference in metabolic activity was observed after 7 days of culture on the two yttrium-containing formulations. Surface roughness has been shown to play a conductive role in the initial cellular adhesion and may explain the difference in metabolic activity at day 2.^47^ The durable nature of the yttrium-containing microspheres highlighted that minimal changes to the material surface integrity and a stable pH of the local microenvironment (Figure 4F) facilitated a suitable environment for cell adhesion. The addition of ions, such as titanium and iron, is commonly employed to increase the durability of phosphate-based glasses to increase cell adhesion and provide a more stable surface to support cell proliferation and differentiation.^48^ While the material surface is vital for cell adhesion and colony establishment, it is not the sole determinant of subsequent biological processes, as the degradation products have the potential to influence the proliferation and differentiation of the cells.^49^

Cells grown on P40, 30Y, and 50Y microspheres all had increased ALP activity in comparison to TCP after 7 days of culture. The increased ALP activity of cells cultured on the microspheres suggested that microspheres provide a more favorable surface for influencing osteoblast cell differentiation than TCP. It was likely that the phosphate, calcium, magnesium, and sodium ions released from the microspheres were stimulating an early osteogenic response. Studies have established that Ca^2+^ ions are required to promote osteocalcin expression and matrix mineralization, while certain concentrations of phosphate and Mg^2+^ added to cells in culture can further induce mineralization.^50^ This is particularly important for bone repair applications, as the formation of a mineralized bone matrix is essential for the restoration of bone structure and function. The lower ion release rates from the yttrium-containing microspheres could lead to a more controlled and sustained release of therapeutic ions, supporting prolonged osteogenic activity and stability in the bone microenvironment. Cells grown on TCP were not exposed to these additional ions, which likely accounts for their significantly lower ALP activity. To comprehensively understand the biological implications of the microspheres’ stability and ion release profiles, future longer-term cell culture studies would also be beneficial. This would allow for evaluation of the impact of sustained ion release and surface integrity on bone cell activity, differentiation, and the overall mineralization process over extended periods.

The spherical geometry of the microspheres allows for a more even distribution of cells around the biomaterial in comparison with irregular-shaped materials. This provides a more consistent microenvironment for cell growth and differentiation, which is crucial for studying tissue regeneration. The surface topography of biomaterials has also been shown to directly influence cellular responses, including adhesion, proliferation, and osteogenic differentiation.^51^ Rough surfaces of native bones mineralized extracellular matrix (ECM) have been identified as an important feature that promotes adhesion and differentiation of osteoprogenitor cells to an osteogenic lineage.^52^ A rough topography, therefore, can effectively mimic the mineralized ECM interface that cells adhere in vivo when participating in bone remodeling and regeneration. The increased ALP activity seen in cells grown on the 30Y and 50Y microspheres, in comparison to the P40 microspheres, may be due to the surface roughness and topographies, although this warrants further study.

The current study emphasizes the prospect of yttrium-containing calcium phosphate glass-ceramics as materials to facilitate bone repair. The prospect of neutron activating the yttrium-containing microspheres demonstrates their capability beyond bone regeneration and may be used to deliver localized radiotherapy at the site of delivery. The novel microsphere formulations satisfy the necessary requirements for internal radiotherapy applications in that they (i) contain high levels of radionuclide within their structure capable of delivering therapeutic radiation and (ii) are chemically durable and resistant to physiological fluids to prevent substantial radionuclide release during a period of radioactivity. Additionally, the yttrium-containing microspheres were cytocompatible and supported osteoblast-like cell growth and proliferation. Microspheres with the capacity to promote bone regeneration and following radiotherapy delivery surpass the utility of glass microspheres solely intended for radiotherapy, offering a comprehensive and integrated therapeutic strategy that addresses both oncological and regenerative needs.

## Conclusions

In summary, this study successfully developed high yttrium-content phosphate-based glass-ceramic microspheres via flame spheroidization for potential applications in bone cancer radiotherapy treatment and bone regeneration. The flame spheroidization process yielded uniform microspheres when sieved into their desired size range of 45–125 μm and allowed for control over yttrium content, ranging from approximately 1–39 mol %. EDX analysis confirmed the homogeneous distribution of yttrium and other elements throughout the microspheres, indicating a uniform composition. XRD analysis revealed the presence of crystalline phases, indicative of glass-ceramic materials formation.

Upon the addition of high yttrium content to phosphate glass, the material transitions from incorporating all of the yttrium into the network to forming crystalline yttrium-rich phases, such as YPO4, within the glass-ceramic microspheres. This resulted in improved chemical durability and resistance to degradation, which are crucial for internal radiotherapy applications. Ion release studies demonstrated reduced release rates of all ions from yttrium-containing microspheres compared with P40 glass microspheres. There was a significant decrease in the release rate of yttrium ions with increasing amounts of yttrium ions incorporated into the microspheres. In vitro cytocompatibility studies revealed favorable cellular responses, supporting the potential of the yttrium-containing microspheres for bone regeneration applications.

The high yttrium content in the microspheres offers the advantage of enhanced radiation delivery per dose, potentially reducing the number of microspheres required for treatment. The ability to administer these microspheres via minimally invasive procedures further enhances their utility in internal radiotherapy applications. Additionally, the enhanced chemical durability ensures that the microspheres remain stable, preventing yttrium leaching and undesired irradiation of surrounding tissues, thereby ensuring that localized and sustained therapeutic effects are delivered over the required treatment period.

Overall, the novel high yttrium-content phosphate glass-ceramic microspheres presented in this study offer a promising dual-purpose platform for combined bone cancer radiotherapy treatment and bone regeneration. Their ability to deliver localized radiotherapy while supporting bone tissue regeneration represents a significant advancement in the field of biomaterials for oncological and regenerative medicine applications. Further in vitro and in vivo studies are warranted to explore their efficacy in preclinical and clinical settings.

## Data Availability

Data will be made available on request to the corresponding author.
